# Editorial: New insights into the genetic mechanisms of thermophilic archaea

**DOI:** 10.3389/fmicb.2023.1185784

**Published:** 2023-04-04

**Authors:** Zhenfeng Zhang, Salvatore Fusco

**Affiliations:** ^1^State Key Laboratory of Microbial Resources, Institute of Microbiology, Chinese Academy of Sciences (CAS), Beijing, China; ^2^Biochemistry and Industrial Biotechnology Laboratory, Department of Biotechnology, University of Verona, Verona, Italy

**Keywords:** thermophilic archaea, archaeal virus, chromosome organization, DNA replication, DNA repair, DNA recombination, RNA transcription

*Archaea* were recognized as the third domain of cellular life, besides *Bacteria* and *Eukarya*, by Carl Woese and George Fox in 1977 (Woese and Fox, 1977). Since then, it has been widely proved that archaea share common features with bacteria in many metabolic aspects, whereas their machineries of DNA transactions resemble those of the eukaryotic counterpart. Indeed, archaeal enzymes and regulatory proteins that constitute the DNA transactions machineries are usually simpler eukaryotic-like versions (Greci and Bell, 2020; Koonin et al., 2020). Taking advantages of the *in vitro* stability of their proteins/enzymes as well as of the design of powerful genetic manipulation tools, thermophilic archaea have established as simpler prokaryotic model systems to explore the complexity of eukaryotic genetic mechanisms.

Most archaeal species encode one or more copies of archaeal histone, which wraps DNA in a similar manner as the histone octamer in eukaryotes (Levin et al., 2018). Indeed, the genomic DNA is kept as a highly compacted structure, yet accessible to the molecular machines functioning in DNA transactions. For instance, the helical polymerization of the nucleosome-like structures may be critical for regulation of gene expression in all histone-encoding archaea. Proofs of this come from a study by Sanders et al., in which mutations were introduced into the histones of *Thermococcus kodakarensis* to block the formation of tightly packed chromatin structures and then the changes in gene expression were quantified. The altered chromatin landscape and the resultant global transcriptome changes revealed the regulatory importance of higher-order histone-based chromatin architectures in archaeal gene expression. It also should be noted that most species of creanarchaea encode Cren7 instead of archaeal histone, which compacts DNA through bending and bridging (Zhang et al., 2020). In *Sulfolobus*, Cren7 undergoes extensive methylations at multiple lysine residues to various extents (Guo et al., 2008), the function of which remains unclear. Ding et al. showed that, unlike for Sis7d, methylation significantly influences the DNA binding, supercoiling, and bridging capacities of Cren7 from *Sulfolobus islandicus*. The conservation of the methylation sites among Cren7 homologs suggested the adoption of a potential eukaryotic-like epigenetic mechanism involved in the chromosomal regulation in crenarchaea.

All living organisms have evolved a variety of DNA repair mechanisms to maintain their genome integrity in response to endogenous and external sources of DNA damage agents. Hyperthermophilic archaea (HA), thriving in high-temperature environments, must face the severe challenge of the accelerated rates of deamination of base in DNA. Lin et al. provided a clear review of the molecular mechanisms involved in the repair of hypoxanthine in DNA, which is operated by DNA glycosylases and endonucleases from HA, and proposed future research directions. Abasic sites are the most abundant DNA lesions in archaeal cells, the replication of which requires specialized DNA polymerases. Feng et al. found that the sequential actions of two polymerases (i.e., Dpo4 and Dpo2 from *S. islandicus*) efficiently promote the bypass of abasic sites. Dpo4 starts the translesion DNA synthesis (TLS) and stalls at +1 to +3 site downstream the lesion, at which Dpo2 efficiently catalyzes further DNA synthesis past the lesion. Archaea also employs the restriction–modification (R-M) system to protect against invading DNA, such as that of viral origin. *Thermococcus kodakarensis* encodes two putative R-M systems (i.e., TkoI and TkoII) that are large multifunctional proteins exerting both methyltransferase and endonuclease activities. Zatopek et al. demonstrated that *T. kodakarensis* strains deleted with either or both R-M enzymes grew more slowly but displayed significantly increased competency compared to the parent strain. The recognition sequences for the methyltransferase and endonuclease in TkoI and TkoII were also identified.

Molecular mechanisms of the biological conflicts and self vs. non-self recognition in archaea remain poorly understood. By phylogenomic analysis, Makarova et al. identified a hypervariable gene module widespread among *Thermococcales*, named VARTIG (VARiable *Thermococcales* IG). The modules consist of an upstream gene encoding a large protein containing several immunoglobulin (Ig) domains and combinations of downstream genes, some of which also contain Ig domains, and showing an overall organization similar to the organization of Polymorphic Toxin Systems (PTS). The functions of VARTIG remain unclear but the identified features of this system imply its potential roles in the inter-microbial conflicts, innate immunity, and self vs. non-self discrimination.

All the articles in this topic provide new insights into the genetic mechanisms of thermophilic archaea and may help us to understand DNA transactions in eukaryotes. The perspectives and hypotheses raised by the authors also provide valuable clues to the mystery of evolution of the three domains of life.

## Author contributions

ZZ and SF wrote and edited the manuscript. Both authors contributed to the editorial and approved the submitted version.
